# Advancing equity, diversity, inclusivity, and accessibility in primary care: The development of an integrated educational experience model

**DOI:** 10.1177/08404704241264236

**Published:** 2024-07-24

**Authors:** Cassandra Barber, L. Jayne Beselt, Jade Alcantara, Bizav Jaffer, Kelly Bute-Seaton, Wendy Chong, Tamara Carver, Heather MacNeill, Bukola Salami, Lyn K. Sonnenberg, J. Cristian Rangel, Constance LeBlanc, Kannin Osei-Tutu, Aimée Bouka, Arun Radhakrishnan, Jerry M. Maniate

**Affiliations:** 1 152971 Bruyère Research Institute, Ottawa, Ontario, Canada.; 2 12367 McGill University, Montreal, Quebec, Canada.; 3 233846 University of Toronto, Toronto, Ontario, Canada.; 4 70401 University of Calgary, Calgary, Alberta, Canada.; 5 3158 University of Alberta, Edmonton, Alberta, Canada.; 6 12365 University of Ottawa, Ottawa, Ontario, Canada.; 7 12361 Dalhousie University, Halifax, Nova Scotia, Canada.

## Abstract

This article presents the development of the Equity, Diversity, Inclusivity, and Accessibility (EDIA) Cross-Cutting Theme Project within the Team Primary Care (TPC) initiative, aimed at addressing systemic inequities through innovative educational strategies. Grounded in the social accountability of health professions framework, this project aims to equip primary care teams with the knowledge, skills, and attitudes necessary to promote health equity. The EDIA Integrated Educational Experience (IEE) model includes a self-assessment tool, digital learning space, and national mentorship network, providing a comprehensive approach for primary care teams to promote health equity. The IEE model utilizes a layered micro, meso, and macro approach to support cultural transformation within highly complex healthcare environments. Key lessons learned involve trust- and relationship-building processes to help dismantle historical silos and encourage open dialogue. Future efforts focus on implementation, ensuring adaptability, scalability, and sustainability, positioning the model as a catalyst for equitable primary care delivery.

## Introduction

Healthcare inequities and accessibility challenges persistently lead to poor health outcomes and significant barriers in healthcare delivery globally.^1-4^ Healthcare systems worldwide face severe distress due to health workforce crises, rapidly changing population needs, complex health challenges, the aftermath of the COVID-19 pandemic, and growing societal demands and expectations.^5-7^ For populations impacted by racism and oppression, these stressors compound existing inequities, rendering their health and care more vulnerable and exacerbating poor outcomes.^8^

Health leaders face significant challenges in addressing equity within health systems. One key issue is the paucity of educational resources, training, and capacity-building for healthcare providers to effectively address equity concerns. Existing educational efforts are often fragmented and siloed, with limited coordination and integration across different healthcare professions, institutions, and regions.^9^ This fragmentation often hinders the development of a comprehensive approach to equity education and training. Additionally, primary care providers often receive limited or no formal training in cultural humility, implicit bias, and trauma-informed care required to provide equitable and inclusive care.^10^

Health inequities, systemic racism, and discrimination are often deeply rooted in complex social, economic, and political factors, requiring a multifaceted and intersectional approach to address them effectively.^11^ Primary care providers must navigate these complexities while addressing diverse population health needs.^12,13^ Addressing these challenges requires a comprehensive and multifaceted approach that targets the root causes and fosters inclusive environments. This article showcases the development of the Equity, Diversity, Inclusivity, and Accessibility (EDIA) Cross-Cutting Theme Project within the Team Primary Care (TPC)—Training for Transformation initiative. Grounded in the social accountability mandate of health professions programs, this project aimed to develop a model to equip primary care teams with the knowledge, skills, and attitudes needed to understand and address healthcare inequities, dismantle systemic barriers, and promote health equity through innovative educational strategies and collaborative co-design processes.

### TPC and EDIA cross-cutting theme project

The TPC pan-Canadian initiative aimed to enhance interprofessional comprehensive primary care capacity through improved training for health leaders and practitioners, fostering transformative collaboration and supporting interprofessional teams.

Within the larger TPC initiative, the EDIA Cross-Cutting Theme, designed and led by the Equity in Health Systems (EqHS) Lab,^14^ aimed to develop a model to better support primary care teams and educators in developing foundational competencies to address pervasive healthcare inequities, discrimination, and exclusion as an intermediate step towards achieving health equity. Additionally, this initiative provided an opportunity to begin to address the current gap in the education and training environment concerning equity in health systems. Being part of the TPC initiative also meant tapping into a broader network of knowledge and resources, fostering collaborations that extend beyond funding to include shared learning and collective impact. This affiliation amplified the project’s reach and significance, enabling the adoption of best practices and innovative solutions across various settings.

### Social accountability framework

The focus of the EDIA project was grounded in the social accountability framework of health professions education.^15^ This framework emphasizes the obligation of health professions programs to direct their education, research, and service activities towards addressing the priority health concerns of the communities they serve.^15^

Social accountability is guided by four core values: relevance, quality, effectiveness, and equity.^15^ This framework mandates that health profession programs address population health needs relevant to their local communities; provide high quality, comprehensive, culturally safe, evidence-based care; utilize cost-effective healthcare resources for maximum public health impact; and strive to promote health equity and universal access.^15^

Embodying these principles, the EDIA project’s objective was to promote equity by dismantling systemic barriers and fostering inclusive environments while designing a model to support primary care teams in developing the knowledge, skills, and attitudes needed to understand their contexts through humility, critical self-reflection, and authentic community engagement. The socially accountable approach aimed to address the inequities and challenges impacting patients, society, and those working and learning within the healthcare system.

Drawing upon these core values, the EDIA project ensured that the educational initiatives and resources developed remained relevant to the specific needs of local communities, provided high-quality and culturally safe content, utilized effective and cost-efficient methods, with the ultimate aim of promoting health equity by addressing systemic barriers and fostering inclusive and accountable environments.

### Developmental evaluation

To capture the process and learnings from this complex, evolving initiative, an overarching developmental evaluation model was employed. Developmental evaluation is designed for initiatives operating in dynamic and uncertain environments, as it facilitates continuous quality improvement, adaptation, and learning throughout the implementation process.^16^ The goal of this continuous evaluation strategy was to identify key insights and learnings that could inform the further development and refinement of the EDIA project, as well as guide future initiatives aimed at promoting health equity in complex healthcare settings. Using the developmental evaluation model, the EDIA Theme exemplifies the principles of social accountability by remaining responsive, adaptive, and accountable to the evolving needs and contexts of primary care teams and the local communities they serve.

## EDIA integrated educational experience model

This project utilized a novel approach by developing the EDIA Integrated Educational Experience (IEE) Model. This model incorporated an educational design framework, unique values-based approach (based upon gratitude, humility, curiosity, listening, reflection, unlearning, and relearning), and operationalized three interconnected components (depicted in Box 1): (1) integrating humility-based insights; (2) tailored learning experiences; and (3) practical application and mentorship.


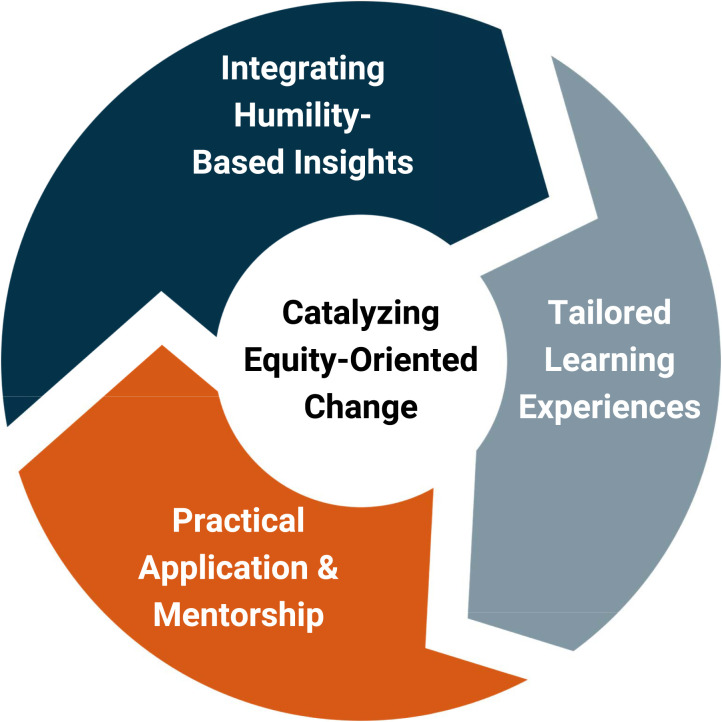


**Figure 1.**
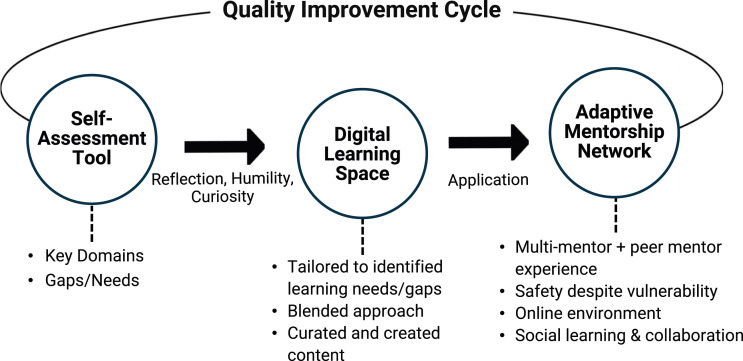
IEE model, designed to advance EDIA in primary care. The model includes three successive components: self-assessment tool, digital learning space, and canadian EDIA adaptive mentorship network. The arrows in the figure represent the flow and integration between these components, illustrating how the self-assessment tool informs the tailored learning experiences within the digital learning space, and how these experiences are further reinforced and applied through the mentorship network. This interconnected approach supports continuous learning, cultural transformation, and the promotion of health equity in primary care settings.

The IEE model is comprised of three successive components: (1) self-assessment tool; (2) digital learning space; and (3) canadian EDIA adaptive mentorship network (depicted in Figure 1).

### Self-assessment tool

The first component of the IEE model is an evidence-informed self-assessment tool designed to help primary care health professionals identify their educational opportunities and areas for growth related to EDIA domains. This self-reflective tool guides participants through a series of indicators across six core EDIA domains (shown in Table 1), identified through a narrative review of journal articles and professional regulatory guidelines. A thematic synthesis was used to identify a common set of indicators across included articles and documents. Upon completion, participants receive personalized recommendations for educational resources tailored to their identified needs, allowing for a customized learning experience focused on addressing self-identified specific areas for improvement.

**Table 1.** EDIA IEE model domains.

1.	Access to care
2.	Anti-racism and discrimination
3.	Accessibility, disability, justice, and anti-ableism
4.	Indigenous health
5.	Health promoting working and learning environment
6.	Leadership development to promote anti-oppression

### Digital learning space

The second component of the IEE model is a curated digital platform that provides comprehensive educational content and resources related to the identified six EDIA domains. This digital space offers self-paced learning opportunities to allow flexibility, enabling participants to engage with the material in a flexible manner. The digital learning space is designed to deepen participants’ knowledge and skills in addressing EDIA issues within the primary care context. It serves as a centralized educational repository for accessing relevant educational materials, interactive modules, and multimedia resources developed through a collaborative co-design process.

### Canadian EDIA adaptive mentorship network

The final component of the IEE model is the Canadian EDIA adaptive mentorship network, which connects participants with experienced mentors from across the country. This network facilitates the practical application of acquired EDIA knowledge and skills through local projects and initiatives aimed at advancing EDIA within participants’ respective contexts. The current network consists of 11 primary care leaders located across the country. These mentors possess expertise in EDIA and aim to provide guidance and support to participants as they develop and implement EDIA-focused initiatives tailored to their primary care settings. The adaptive mentorship model has been successfully used in clinical settings to build capacity in primary care^17^ and was modified to fit the specific needs of this project.

Participants who complete all components of the EDIA IEE model will be equipped with an approach grounded in concepts, competencies, resources, and guidance to effectively address EDIA issues and promote health equity within their unique primary care contexts, practices, and communities.

### Content validity

All components of the IEE model were validated using one-on-one consultations with primary care leaders with expertise in EDIA, individuals with lived experiences, an Indigenous advisory leadership group, and expert panel reviews. The expert panel consisted of up to 30 experts, including academic scientists, primary care leaders, and measurement experts. These consultations and reviews ensured that the context designed in each component of the model was conceptually valid and relevant to the needs of primary care health professional and patients.

## Discussion: Digging deeper

The development of the IEE model required a comprehensive and collaborative approach, guided by the four core social accountability values aimed at addressing systemic inequities in primary care.

### Building collaborative partnerships and processes

The simultaneous development of the IEE model’s interconnected self-assessment tool, digital learning space, and adapted mentorship network necessitated the creation of several working groups and advisory committees comprised of primary care health professionals, Indigenous elders, academic scientists, program evaluators, educational technologists, patient partners, individuals with lived experiences, and learners. These broad advisory committees ensured diverse representation from various primary care leaders within the healthcare system. This collaborative co-design process was essential for tackling the complex nature of EDIA initiatives, fostering psychological safety, and ensuring diverse perspectives were incorporated.

The intentional inclusive design facilitated a collaborative co-design process, enriching the outputs with a wide range of perspectives and expertise. Trust-building and relationship-building processes played integral roles in overcoming historical and ongoing silos and creating safe spaces for open dialogue on sensitive EDIA topics. Trust-building facilitated the partnership and implementation of strategies intended to address healthcare delivery inequities.

Layered structures and broad engagement of individuals with expertise at multiple levels (micro-, meso-, macro-) were necessary to support cultural transformation within highly complex healthcare environments. This involved engaging subject matter experts, patient partners with lived experiences, measurement experts, and other stakeholders in interconnected working groups and advisory committees.

The iterative development process allowed for real-time feedback, evaluation, and adaptation to the evolving needs and context of primary care teams and communities. This necessitated regular reflective evaluation meetings with the development team, primary care partners, and community stakeholders to begin to assess the potential effectiveness, relevance, and future impact of the IEE model. These reflective evaluations provided valuable insights and opportunities for continuous quality improvement, ensuring that the IEE model remained responsive and aligned with the dynamic needs of primary care teams and the communities they serve.

### Aligning the IEE model with social accountability framework

The EDIA IEE model closely aligns with the social accountability mandate of health professions education, emphasizing the importance of addressing priority health needs, engaging communities and partnerships, promoting health equity, ensuring continuous improvement, and adopting a comprehensive systems-based approach. The IEE model aims to empower primary care teams with the knowledge, skills, and attitudes needed to understand and address the priority health needs of marginalized communities affected by systemic inequities. By advancing EDIA competencies, primary care health professionals can deliver more relevant, culturally safe, and equitable services tailored to the specific needs of local populations.

Authentic engagement with communities to co-identify their health priorities is a fundamental principle of social accountability.^15^ The IEE model facilitates this by involving patient partners and individuals with lived experiences in the co-design process, curriculum development, and advisory committees. This community partnership approach ensures that educational content and initiatives remain relevant and responsive to priority community health needs. Striving to achieve better health equity is a central goal of social accountability. The IEE model directly aligns with this mandate by aiming to dismantle systemic barriers, challenge discriminatory practices, foster inclusive environments, and ultimately reduce health disparities through its EDIA-focused education, and mentorship.

The developmental evaluation approach provided continuous feedback and iterative improvements which were critical in refining the IEE model. This approach allowed adaptation, responsiveness, and accountability to evolving community needs, ensuring the model’s relevance in various contexts. An important aspect of our evaluation approach was to ensure objectivity by intentionally not involving the evaluator in the development of the IEE model. This design allowed the evaluator to remain unbiased and focused on capturing essential insights and learnings. Additionally, one-on-one consultations and expert panel review used to validate the model’s context was necessary to ensure relevance in practice.

### Lessons learned for health leaders

Key practical implications for health leaders included creating safe environments for dialogue, fostering a culture where team members felt comfortable expressing their views and experiences. This was vital for addressing and overcoming systemic biases and inequalities. Clear role delineation was critical for defining and communicating roles and responsibilities to ensure effective collaboration and accountability. When team members understood their specific duties and expectations, they worked more cohesively and effectively towards common goals. Ensuring diverse representation in the development processes was essential for fostering inclusivity. This included diverse perspectives, such as those of patient partners, Indigenous advisory team, and individuals with lived experiences, to enrich the decision-making process. Incorporating a wide range of viewpoints, health leaders can develop more comprehensive and effective strategies to address the needs of carious communities.

Reflective evaluations were implemented continuously to identify areas for improvement and adapt to evolving needs. This ongoing process allowed stakeholders to assess the impact of their initiatives, learn from their experiences, and make necessary adjustments. Utilizing interconnected working groups and advisory committees promoted board engagement and collective problem-solving by bringing together individuals with different expertise and experiences. This collective approach ensured that initiatives were well-rounded and more likely to succeed.

The developmental evaluation approach highlighted several successes and areas for improvement. One key success was the iterative feedback process, where regular feedback from stakeholders helped refine the model components, ensuring they remained relevant and effective. This ongoing input was crucial for making timely adjustments and enhancements to the model. Adaptability was another significant strength of the model, as it was continuously adapted based on participant input. This flexibility made the model suitable for diverse primary care settings, allowing it to be tailored to meet the specific needs of different environments and populations.

Insights from the developmental evaluation also informed practical steps for implementing the model. For instance, integrating humility-based insights into team dynamics helped create a more inclusive and reflective practice environment. Additionally, creating tailored learning experiences equips primary care health professionals with the knowledge needed to better understand the systemic inequities within the healthcare system.

## Conclusion

This project highlights the importance of intentional and broad engagement and layered structures in developing EDIA initiatives across healthcare systems. The EDIA IEE model offers a comprehensive approach that may lead to cultural transformation in clinical practice, health professions education, and the broader health system.

The development of the EDIA IEE model provides health leaders with a flexible framework to promote equity, diversity, inclusion, and accessibility. Key practical implications include fostering safe environments for dialogue, clear role delineation, and diverse representation in development processes, despite time constraints. Interconnected working groups and advisory committees, including patient partners, ensure inclusivity. Reflective evaluations support continuous improvement and adaptation to evolving needs. Championing the adaptability of the EDIA IEE model enables health leaders to scale this approach to diverse settings, with the potential to driving meaningful impact in promoting health equity.

Future research will involve piloting the IEE model across various primary care settings within Canada’s publicly funded healthcare system over the next 12 months. The piloting phase will include comprehensive pre- and post-implementation evaluations to measure EDIA competencies of primary care health professionals and interprofessional teams and the experiences of patients receiving care. These evaluations will provide valuable data on the model’s effectiveness in enhancing the competence of primary care providers and improving the experiences of patients in receiving such care. Evaluation outcomes can also be used to inform necessary adjustments and enhance the model’s impact, offering practical feedback for health leaders interested in implementing the model in their context.

The complexity identified underscores the challenges in developing EDIA initiatives, suggesting the importance of building on existing efforts and adapting to local needs. As the TPC project funding concludes, efforts will shift to implementation, sustainability, and scaling beyond primary care. The model’s inherent adaptability allows for its extension to national and international health systems, as well as beyond the healthcare sector. Health leaders are urged to champion these components, recognizing their potential to drive positive change and empower health professionals to address complex challenges and impact communities.

## References

[1] Friel S Marmot MG . Action on the social determinants of health and health inequities goes global. Annu Rev Publ Health. 2011;32(1):225-236. doi:10.1146/annurev-publhealth-031210-101220. 21219162

[2] World Health Organization . Global report on health equity for persons with disabilities. 2022. https://www.who.int/publications/i/item/9789240063600. Accessed April 5, 2024.

[3] Gréaux M Moro MF Kamenov K , et al. Health equity for persons with disabilities: a global scoping review on barriers and interventions in healthcare services. Int J Equity Health. 2023;22(1):236. doi:10.1186/s12939-023-02035-w. 37957602 PMC10644565

[4] Shadmi E Chen Y Dourado I , et al. Health equity and COVID-19: global perspectives. Int J Equity Health. 2020;19(1):1-16. doi:10.1186/s12939-020-01218-z. PMC7316580 32586388

[5] Aymerich C Pedruzo B Pérez JL , et al. COVID-19 pandemic effects on health worker’s mental health: systematic review and meta-analysis. Eur Psychiatr. 2022;65(1):e10. doi:10.1192/j.eurpsy.2022.1. PMC8828390 35060458

[6] World Health Organization . Global strategy on human resources for health: workforce 2030. 2020. https://iris.who.int/bitstream/handle/10665/250368/?sequence=1. Accessed April 5, 2024.

[7] Lakin K Kane S . Peoples’ expectations of healthcare: a conceptual review and proposed analytical framework. Soc Sci Med. 2022;292:114636. doi:10.1016/j.socscimed.2021.114636. 34894457

[8] Fanta M Ladzekpo D Unaka N . Racism and pediatric health outcomes. Curr Probl Pediatr Adolesc Health Care. 2021;51(10):101087. doi:10.1016/j.cppeds.2021.101087. 34711499

[9] Health Canada . Certain circumstances issues in equity and responsiveness in access to health care in Canada. 2001. https://www.canada.ca/content/dam/hc-sc/migration/hc-sc/hcs-sss/alt_formats/hpb-dgps/pdf/pubs/2001-certain-equit-acces/2001-certain-equit-acces-eng.pdf. Accessed April 5, 2024.

[10] Garrod M Vafaei A Martin L . The link between difficulty in accessing health care and health status in a Canadian context. Health Serv Insights. 2020;13. doi:10.1177/1178632920977904. PMC7727055 33343198

[11] Gibbs T . Sexy words but impotent curricula: can social accountability be the change agent of the future? Med Teach. 2011;33(8):605-607. doi:10.3109/0142159X.2011.590251. 21774644

[12] Stange KC Jaén CR Flocke SA Miller WL Crabtree BF Zyzanski SJ . The value of a family physician. J Fam Pract. 1998;46(5):363-368. 9597993

[13] Starfield B Shi L Macinko J . Contribution of primary care to health systems and health. Milbank Q. 2005;83(3):457-502. doi:10.1111/j.1468-0009.2005.00409.x. 16202000 PMC2690145

[14] Equity in Health Systems (EqHS) Lab. https://www.eqhslab.com/.

[15] Boelen C Heck JE World Health Organization . Defining and Measuring the Social Accountability of Medical Schools. World Health Organization; 1995. https://iris.who.int/bitstream/handle/10665/59441/WHO_HRH_95.7.pdf?sequence=1&isAllowed=y. Accessed April 5, 2024.

[16] Patton MQ . Developmental Evaluation: Applying Complexity Concepts to Enhance Innovation and Use. New York, NY: Guilford Press; 2011.

[17] Radhakrishnan A Clarke L Greenberg L . How collaborative mentoring networks are building capacity in primary care. Healthc Q. 2018;22(3):54-60. doi:10.12927/hcq.2019.26016. 31845859

